# The palatal displaced maxillary canine: early diagnosis and interceptive correction - a guideline for the general dental practitioner

**DOI:** 10.1038/s41415-025-8892-z

**Published:** 2025-10-10

**Authors:** Martyn T. Cobourne, Jadbinder Seehra, Spyridon N. Papageorgiou

**Affiliations:** 332451673522687706407 Centre for Craniofacial & Regenerative Biology, Department of Orthodontics, Faculty of Dental, Oral & Craniofacial Sciences, King´s College London, London, United Kingdom; 323507270908392286494 Clinic of Orthodontics and Pediatric Dentistry, Centre for Dental Medicine, University of Zurich, Zurich, Switzerland

## Abstract

The maxillary permanent canine has a long path of eruption and can become displaced, leading to displacement on the palatal side and failure of eruption. We highlight current diagnostic strategies to help monitor eruption of this tooth and discuss potential interceptive measures to promote eruption in the presence of displacement. We further investigate the current evidence base with meta-analysis and provide a guide for the general dental practitioner based upon these data. In particular, extraction of the primary canine to normalise the eruptive position of a palatal displaced maxillary canine is unpredictable, but a favourable outcome can be achieved if treatment is commenced between the ages of 10-13 years, the patient is in the mixed dentition, there is space for the permanent canine within the dental arch, and radiographically, the canine crown is not beyond the midline of the lateral incisor tooth. It is important that the general dental practitioner can diagnose normal and abnormal eruption of the permanent canine in children aged between 10-13 years and refer those cases with evidence of displacement in a timely and appropriate manner.

**Supplementary Information:**

Zusatzmaterial online: Zu diesem Beitrag sind unter 10.1038/s41415-025-8892-z für autorisierte Leser zusätzliche Dateien abrufbar.

## Key points

- Highlights the clinical problem of palatal displaced maxillary canines.
- Discusses appropriate diagnosis for the general dental practitioner.
- Analyses the contemporary data relating to interceptive management of this condition with focus on extraction of the primary canine.
- Meta-analyses the data in an accessible and understandable way.

**Supplementary Information:**

Zusatzmaterial online: Zu diesem Beitrag sind unter 10.1038/s41415-025-8892-z für autorisierte Leser zusätzliche Dateien abrufbar.

## Introduction

The maxillary permanent canine is an important tooth in the dentition, contributing to a robust functional occlusion and playing a significant role in smile aesthetics. This tooth develops high in the maxilla, has a long and time-consuming path of eruption, and can become displaced in buccal or palatal directions with varying degrees of horizontal displacement. The palatal displaced maxillary canine (PDC) often fails to erupt and will require multidisciplinary management, involving surgical intervention and orthodontic treatment. This treatment can be prolonged and associated with complications, including local non-eruption of the canine and tooth resorption.^1^^,^^2^ Overall, the management of a PDC can represent a significant burden for the patient, particularly when the diagnosis is made late and treatment extends into the late teenage years. It is for these reasons that early identification of the PDC is important, allowing the instigation of potential interceptive procedures but also ensuring that, if required, definitive treatment can be undertaken in a timely manner. It is the general dental practitioner (GDP) who is most likely to first encounter the patient with a PDC and here, we highlight the importance of early diagnosis, appropriate referral, and the current evidence base relating to simple interceptive management.

## Assessing the maxillary permanent canine in the developing dentition

As part of routine clinical assessment and monitoring of the developing dentition in children and teenagers, the GDP should be mindful of the maxillary permanent canine and in particular, appropriate development and eruption of this tooth (Table 1).^3^ In children under the age of ten years, the maxillary canine cannot always be palpated in the buccal sulcus and routine radiographic assessment is not usually required at this time.^4^ However, any family history of PDC,^5^ agenesis, or the presence of diminutive/peg-shaped maxillary lateral incisors^6^^,^^7^ should heighten awareness of the potential for a PDC. Once a child has reached the age of ten years, the maxillary canines should be palpable in the buccal sulcus for the majority of children and clinical examination should be an essential part of the routine dental assessment carried out in this age group by the GDP. If the canines cannot be palpated at his stage, or there is asymmetry of palpation or eruption of these teeth, then radiographic investigation is advised.^8^

**Table 1:** Checklist for the assessment of unerupted maxillary permanent canines^3^

**Assessment of unerupted maxillary permanent canines in the mixed dentition**	
History	Family history of palatal displaced maxillary canine; agenesis or diminutive maxillary lateral incisors
Inspection	Assess the presence and appearance of the maxillary lateral incisor (absent, diminutive or peg-shaped) Assess bulge of unerupted canine in relation to adjacent lateral incisor: Crown of lateral incisor proclined (canine buccally positioned) Crown of lateral incisor retroclined (canine palatally positioned) Crowding of the upper dental arch
Palpation	Palpate the buccal sulcus and palatal vault Assess the mobility of the retained primary maxillary canine Assess the mobility of the adjacent lateral incisor
Radiographs	If palpation suggests the maxillary permanent canine is in an abnormal position or it cannot be palpated, then radiographs will be required to determine the position

## Radiographic assessment of the maxillary permanent canine in the developing dentition

**Fig. 1:**
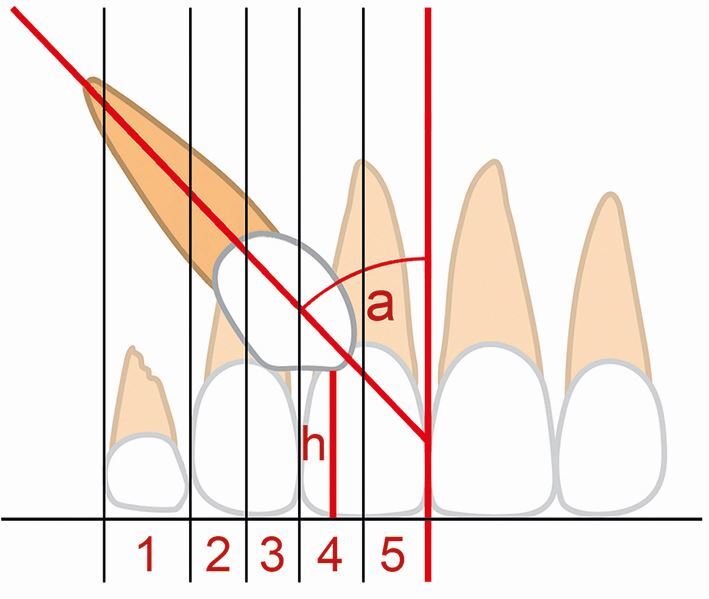
Three criteria for assessing the position of the maxillary canine tooth on a radiograph. 1st criterion: horizontal position of the canine crown in relation to a vertical line drawn between the maxillary central incisors (mid-sagittal plane/midline) and based upon five vertical sectors: sector one, primary canine; sectors two and three, bisecting the midline of the maxillary lateral incisor (distally and mesially, respectively); and sectors four and five, bisecting the midline of the maxillary central incisor (distally and mesially, respectively). 2nd criterion: alpha angle (a), the angulation of the long axis of the canine to a vertical line drawn between the maxillary central incisors (mid-sagittal plane). 3rd criterion: height (h) of the canine cusp tip to the occlusal plane. The higher the sector position (particularly beyond sector two), the greater the alpha angle and higher the canine, the poorer the prognosis for eruption

For the majority of GDPs, periapical, upper standard occlusal, or dental panoramic radiography are readily accessible in the practice environment and these images can all provide essential information on development of the dentition, particularly the permanent maxillary canine.^9^ Three criteria have been proposed to assess the position of this tooth on a panoramic radiograph (Fig. 1): 1) horizontal position of the canine crown in relation to a vertical line drawn between the maxillary central incisors (mid-sagittal plane/midline) and based upon five vertical sectors; 2) angulation of the long axis of the canine to a vertical line drawn between the maxillary central incisors (mid-sagittal plane); and 3) height of the canine cusp tip to the occlusal plane.^10^ Moreover, two films can be used to definitively establish labiolingual position of the maxillary canine through the parallax (or tube-shift) technique.^3^ Horizontal parallax uses a horizontal shift in the x-ray tube (achievable with successive periapical views taken with the tube moved horizontally), while vertical parallax uses a vertical shift in the tube (achievable with a panoramic radiograph and either a periapical or upper standard occlusal view). Cone-beam computed tomography (CBCT) is a further imaging modality that is increasingly being used to locate the position and anatomical relationships of maxillary canine teeth.^11^ However, the routine use of CBCT for the diagnosis of unerupted maxillary canine teeth is not justified because of the significantly increased effective dose compared to intra-oral radiography. CBCT should be prescribed when conventional radiography does not provide adequate diagnostic information, and this will normally be undertaken by the orthodontic specialist.

## Avoid watchful waiting

**Fig. 2:**
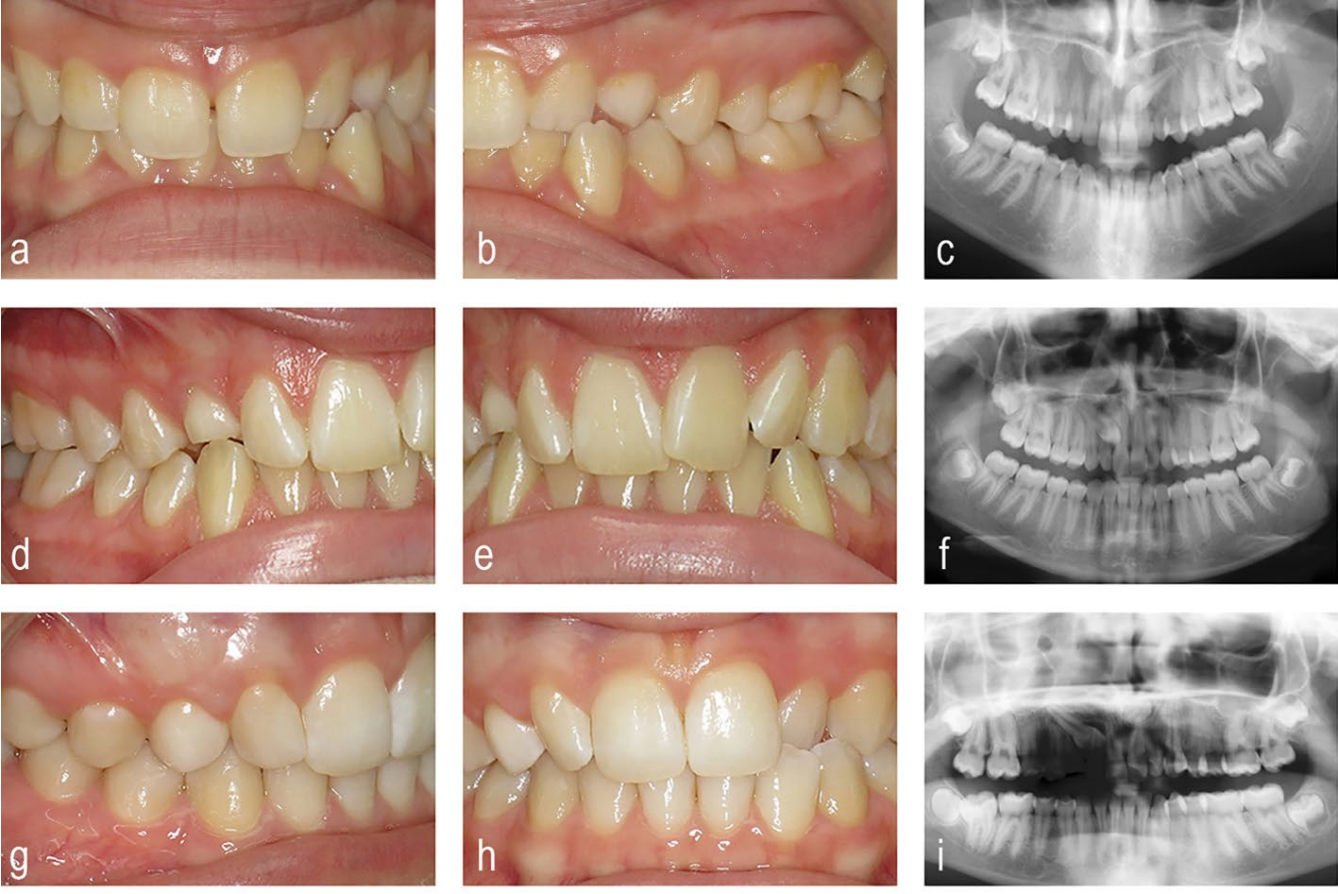
Three seemingly relatively mild malocclusions in female teenage patients, but all three have retained maxillary primary canines and palatal impacted maxillary permanent canines revealed by panoramic radiography. a, b, c) In the upper case, the upper left primary canine is retained with 23 in a mesioangular position close to the midline in sector five. d, e, f) In the middle panels, the upper right primary canine is retained with 13 also mesioangular and towards the midline in sector four. g, h, i) In the lower panels, the the upper right primary canine is retained with 13 also mesioangular but in a slightly more favourable position overlying 12 in sector three; however, there is also an unerupted supernumerary tooth associated with the apex of 21. All three patients had the primary canines extracted, closed exposure of the canines and orthodontic traction to erupt the canines

It is important for the GDP not to be reassured that normal (albeit slow) dental development is taking place in teenagers presenting with firm primary maxillary canines (Fig. 2). This impression can be compounded when primary second molar teeth are still present and there is a seemingly mild malocclusion. Dental development varies significantly with chronological age and the presence of primary molars is not a reason to delay investigation of a non-palpable maxillary permanent canine in teenagers.

## Interceptive management of the palatal displaced maxillary canine

**Fig. 3:**
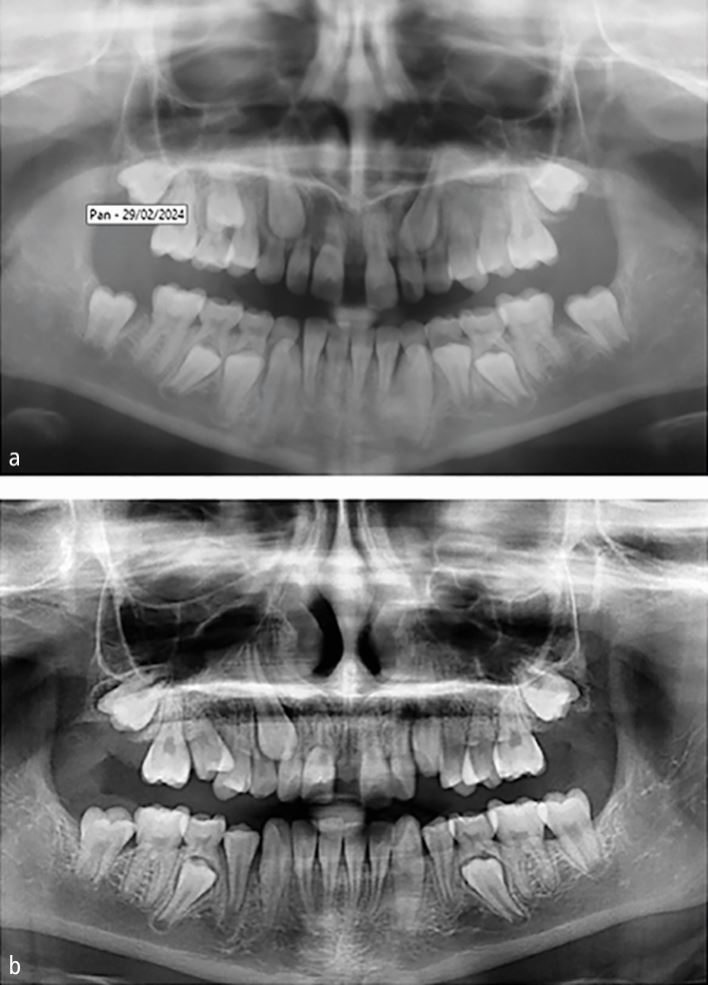
a, b) Interceptive extraction of the upper left primary canine in an 11-year-old patient has been followed by eruption of the palatal displaced sector three 23 over a follow-up period of 12 months (upper and lower panels, respectively). In contrast, the buccal palpable 13 has remained unerupted. Does interceptive extraction of the maxillary primary canine help facilitate eruption of the permanent canine or simply speed the process up?

Early identification of an abnormal developmental position in relation to the maxillary permanent canine and the instigation of interceptive treatment to encourage self-correction can be beneficial for several reasons, including avoiding the need for future surgical exposure and orthodontic traction, reducing the risk of root resorption affecting adjacent maxillary incisor roots, and avoiding potentially prolonged orthodontic treatment. However, the evidence base is equivocal, not of the highest quality, and difficult to interpret. Interceptive treatment for the PDC is based primarily upon the principle of space being available in this region of the maxillary arch, which may involve extraction of the retained primary canine only (Fig. 3), or extraction of this tooth combined with orthodontic mechanics to expand or lengthen the dental arch.

## To extract the primary canine or not? What does the evidence suggest?

The most robust current systematic review assessing the effectiveness of interventions to intercept and prevent a PDC has rated the evidence as very uncertain.^12^ The lack of high-quality evidence has been attributed to poor trial conduct and reporting, variation in study designs, including inconsistent eligibility criteria, variation in primary outcomes, inclusion of both unilateral and bilateral PDCs in mixed parallel and split-mouth designs, lack of control groups, variation in the definition of ‘successful eruption' and the inclusion of participants with varying degrees of horizontal canine displacement and angulation.

Historically, justification for interceptive extraction of the primary canine stems from the findings of a single retrospective cohort study from Sweden involving 35 patients aged between 10-13 years of age. The authors reported successful eruption of 83% suspected PDCs following removal of the primary canine.^10^ However, the absence of an untreated control group and a lack of sample homogeneity in relation to PDC diagnosis (particularly with regard to midline position of the canine crown) make the reported findings susceptible to bias. A similar retrospective study conducted in the United Kingdom some years later and involving 39 patients around the age of 11 years reported the successful eruption of 62% of PDCs following removal of the primary canine. Importantly, a lack of space within the arch adversely affected eruption; however, the lack of an untreated control group and inconsistent sample selection also meant a high risk of bias.^13^

One of the earliest prospective investigations was a three-arm trial led by the late Tiziano Baccetti which included two intervention groups and an untreated control group. Patients with PDCs were allocated to extraction of the primary canine only, extraction of the primary canine combined with cervical pull extra-oral traction to lengthen the dental arch posteriorly, or no treatment. Here, successful eruption of PDCs in the extraction-only and untreated control groups was almost identical at around 50%, but higher in the group where extraction took place in combination with cervical pull headgear (80%).^14^ However, participant allocation to the three groups was unequal and the degree of horizontal displacement was assessed as sector three for all the PDC's - potentially the most favourable position for eruption, if adequate space is available in the arch. Despite being published as a standalone randomised trial, this study was then seemingly repeated with an extended sample, going on to report successful eruption of the PDC in the untreated control group, extraction-only group and extraction-headgear group in 36%, 66% and 88% of cases, respectively.^15^ Concerns regarding the methodology and reporting of these studies have been highlighted^16^ and the results should be treated with extreme caution.

A trial involving participants with bilateral PDCs aged between 10-14 years of age has been undertaken in Sweden and led by Farhan Bazargani.^17^ As part of the methodology, one PDC was allocated to the intervention with the contralateral PDC serving as the control in a sample of 24 participants. The authors reported 62% of PDCs successfully erupting following extraction of the primary canine, while success rate for the control side was 42%. The intervention was more successful in younger participants (aged between 10-11 years) and, as might be expected, when the canine was closer to the normal horizontal position. For example, an improvement in horizontal position of the canine was observed in 15/19 (79%) teeth that were initially in sector two and three. In contrast, only 1/5 (20%) teeth initially in sectors four or five improved. The closer the PDC was to the mid-sagittal plane, the less likely there was an improvement in position following extraction of the retained primary canine. However, the sample size was small, the study design was split-mouth and again, there was a lack of pre-treatment equivalence in terms of sector displacement of the PDC between quadrants in the intervention and control groups.

The most robust investigation relating to primary canine extraction as an interceptive strategy for managing PDC is a two-arm randomised clinical trial performed by Julia Naoumova and colleagues, also in Sweden.^18^ Children aged between 10-13 years of age with unilateral or bilateral PDCs were randomised into primary canine extraction or untreated control groups. Interestingly, 69% of PDCs erupted successfully following extraction of the primary canine compared to 39% of controls. Furthermore, almost twice as many PDCs required subsequent surgical exposure in the control group compared to the extraction group. Although these results seem promising, several factors in the study methodology need to be considered. The study combined unilateral (randomised on an individual basis) and bilateral (randomised on a split-mouth basis) canines, and the PDC observation period for eruption was two years; however, the primary canine was extracted after one year in the untreated control groups if there was no sign of mobility of the primary canine. Additionally, surgical exposure was undertaken in either group after 12 months if there was no radiographic sign of improvement in the position of the maxillary canine. In a follow-up study using CBCT imaging from the sample, the distance of the canine cusp tip to the mid-sagittal plane was reported to be a positive prognostic factor indicating whether the PDC would spontaneously erupt or not.^19^ If the canine cusp was 11 mm from the mid-sagittal plane (midline) then there was a high chance of spontaneous eruption. Conversely, if the cusp was less than 6 mm from the mid-sagittal plane, then surgical exposure would be required, even if the primary canine had been extracted.

## Synthesis of available studies

Here we have identified all available randomised trials in this subject area and synthesised the results via meta-analysis. We identified existing systematic reviews,^12^^,^^16^^,^^20^^,^^21^^,^^22^^,^^23^^,^^24^ checked their reference lists and citations received through Google Scholar, and manually searched Medline (through PubMed) and Google Scholar for relevant primary studies. Included were randomised trials on human patients (both parallel and within-person randomisation) assessing the effect of interceptive extraction of primary canines on the eruption of displaced permanent canines. Secondarily, we included studies of similar design that compared additional extraction of the primary first molar to the extraction of the primary canine alone. Even though the scope of the present paper is focused on PDC, we were more lenient in our search and also included studies with labially or centrally displaced canines (and recorded this transparently).

The literature search yielded a total of five studies comparing interceptive extraction of the primary canine to a control group with no extraction. Four trials were single published papers^15^^,^^17^^,^^25^^,^^26^ and one trial produced multiple papers (and a doctoral thesis).^18^^,^^19^^,^^27^^,^^28^^,^^29^ Three studies were identified comparing extraction of both primary canine and first molar to extraction of only the primary canine and related to four publications.^30^^,^^31^^,^^32^^,^^33^

Considerable variation was seen in the eligibility criteria and patient characteristics of included trials, while in several studies, the extraction of more than one tooth per patient was included and within-patient clustering was not always adequately addressed.^34^ Therefore, the corresponding authors of all included trials were contacted to request their raw dataset^35^ to allow better representation of trial data (see online Supplementary Information Table 1); the only exceptions were the two trials from Tiziano Baccetti who is sadly (deceased).^15^^,^^25^ As a result, two trialists provided at least some parts of their dataset^26^^,^^29^ and one generously provided adjusted-for-clustering estimates for the main outcome.^17^ Generalised linear models for the binomial family were used to take clustering into account and estimate adjusted odds ratios (OR) with their 95% confidence intervals (CI) for eruption of the permanent canine. Additionally, potential confounding factors to eruption of the permanent canine were identified with the change-in-estimate method (with a minimum of 10% change set as cut-off)^36^ and accounted for. Moreover, one trial^26^ included patients aged 7.7-12.0 years who were at risk of maxillary canine impaction, as judged by a canine-to-midline angle of ≥15°.^37^ However, only patients aged ten years or older were included in the present analysis to make the dataset more compatible with data from two studies,^17^^,^^29^ which used the same cut-off of ten years.

Data from similar studies assessing similar outcomes were pooled using meta-analysis with ORs or mean differences (MDs) with their 95% CIs as effect measures. Since treatment effects were expected to vary according to patient-, tooth-, and malocclusion-related characteristics (age, sex, tooth size, tooth position, and space within the dental arch), a random-effects model was deemed *a priori* more appropriate to pool data and calculate the average distribution of treatment effects among the included studies.^38^ A restricted maximum likelihood variance estimator with the Knapp-Hartung correction was used based on relevant guidance.^39^ Between-study observed heterogeneity/estimated relative inconsistency was assessed using the tau^2^/I^2^ metric, respectively, while uncertainty around them was also estimated^40^ for meta-analyses with ≥3 studies. Existing heterogeneity was incorporated into the meta-analytic results with 95% predictions that provide a range of possible outcomes in a future scenario,^41^ again for meta-analyses with ≥3 studies. Meta-analyses were visualised using contour-enhanced forest plots^42^ to assess effect magnitude, imprecision and inconsistency.

## Evidence on extraction of the primary canine

Descriptives for the five included trials are presented in Table 2. Two trials originated from the same research group in Italy,^15^^,^^25^ two trials originated from separate groups in Sweden,^17^^,^^27^ and one from Belgium.^26^ All were of parallel design, except one,^17^ which randomised sides in a split-mouth manner. Apart from extraction of the primary canine, other interventions were sometimes administered (transpalatal arches, headgear, or slow maxillary expansion), but these interventions were ignored in the present analysis, primarily because of the small sample sizes and low-quality of the current evidence base. One trial from Italy^15^ and one from Belgium^26^ included patients considerably younger than ten years of age, but the dataset from the latter was reduced to include only patients aged ten years or older. All included studies included both unilateral and bilateral cases, except for the split-mouth study^17^ which included only bilateral cases. Permanent canines were almost always displaced palatally, except for one study^26^ which also included those in a labial position. Some variation existed among trials in the criteria used to diagnose impaction and included sample sizes ranging from 24-67 patients per trial (48-89 teeth per trial). All patients were followed for 18 months after extraction or up to eruption of the permanent canine.

**Table 2:** Descriptives of included studies

**Trial**	**Design; setting; country†**	**Groups‡**	**Timing criteria**	**UniL/BiL**	**Displacement criteria**	**Other**	**Patients (teeth)**	**Follow-up**
**Extraction III versus control**								
Baccetti, 2008^14^	RCT_PAR_; Uni; ITA	G1: ExIII G2: Ctr *(G3: ExIII+HG)*	8.0-13.0 yrs; CVM<3	UniL/BiL PDCs	OPT / PA (CR)	Cl.II/III tendency; mild ALD	45 (51)	18 mos
Baccetti, 2011^24^	RCT_PAR_; Uni; ITA	G1: ExIII G2: Ctr *(G3: ExIII +TPA)* *(G4: ExIII +TPA+HG)*	9.5-13.0 yrs; late mixed dentition; CVM 1-4	UniL/BiL PDCs	OPT (α≥15⁰)	-	53 (^76)^	Up to permanent dentition & CVM≥5; mean 30 mos
Bazargani, 2014^‡^^16^	RCT_WP_; Clin; SWE	G1: ExIII G2: Ctr	10.0-14.0 yrs; late mixed dentition;	BiL PDCs	Palpation; PA / OPT (sectors 2-5; exceeding lateral axis)	ALD ≤3 mm	24 (^48)^	6/12/18 mos
Naoumova, 2014_coll_^β^^26^	RCT_PAR_; Uni; SWE	G1: ExIII G2: Ctr	10.0-13.0 yrs	UniL/BiL PDCs	Palpation; PA (CR)	ALD ≤2 mm; no RR	67 (^89)^	6/12 mos
Willems, 2023^¥^^25^	RCT_PAR_; Uni; BEL	G1: ExIII G2: Ctr *(G3: SME)*	7.7-12.0 yrs;	UniL/BiL LPDs/PDCs	OPT (α≥15⁰; sector≤1); incomplete root	ALD ≥2 mm; no RR	31 (^59)^	6/12/18 mos
**Extraction III-IV versus extraction III**								
Alessandri Bonetti, 2010^32^	RCT_PAR_; Uni; ITA	G1: ExIII G2: ExIII-IV	9.0-12.6 yrs	UniL/BiL CDCs/PDCs	Palpation or lateral malposition or OPT (α≥25⁰) or OPT (sector>1)	-	59 (^108)^	18 mos
Alessandri Bonetti, 2011^31^	RCT_PAR_; Uni; ITA	G1: ExIII G2: ExIII-IV	8.0-13.0 yrs; CVM<3	UniL/BiL CDCs/PDCs	Palpation or lateral malposition or OPT (α≥25⁰) or OPT (sector>1)	-	37 (^65)^	18 mos
Hadler-Olsen, 2020_coll_^30^	RCT_PAR_; Clin; NOR	G1: ExIII G2: ExIII-IV	9.5-13.5 yrs;	UniL/BiL PDCs	PA (CL); OPT (sector III-IV) or OPT (sector II & α≥25⁰)	-	32 (^48)^	Until eruption
† = Countries given using the ISO-3 coding ^‡^ = Groups in Italic are given experimental groups of the original studies that were disregarded in this analysis ALD = arch length; discrepancyBiL = bilateral; CDC = centrally displaced canine; Cl = Angle's classification; Clin = clinic; _coll_ = collated multiple publications; CR = Clark's rule; Ctr = control group (no treatment); CVM = cervical vertebrae maturation method; ExIII = extraction of the primary canine; ExIII-IV = extraction of the primary canine and the primary first molar; G = group; HG = headgear; LDC = labially displaced canine; mos = months; OPT = orthopantomogram; PA = periapical radiograph; _PAR_ = parallel design; PDC = palatally displaced canine; RCT = randomised clinical trial; RR = root resorption; SME = slow maxillary expansion; TPA = transpalatal arch; Uni = university clinic; UniL = unilateral; _WP_ = within-persons (clustered) design; yr = year.								

Re-analysis of two studies^18^^,^^19^ was performed using the dataset provided (see online Supplementary Information Table 2a). Eruption of the permanent canine was significantly lower when its inclination to the midline increased (and especially when this was ≥30 °), when it was in sector 3 (Fig. 1) or higher, and when its root was more developed (*p* <0.05, in all instances). The effect of these factors seemed to be similar both for unilateral and bilateral cases. When potential identified confounders (see online Supplementary Information Table 2b) were taken into account, extraction of the primary canine was associated with improved eruption odds for the permanent canine overall (OR = 3.5; 95% CI = 1.4-8.9; *p* = 0.008). Interestingly, the effect of primary canine extraction varied considerably for patients with unilateral and bilateral impacted canines (ORs of 2.3 and 5.7, respectively). This could be either a statistical artefact or might indicate systematic differences between unilaterally and bilaterally impacted cases.

Re-analysis of Willems *et al*.^26^ is seen in online Supplementary Information Table 3. When analysing the whole sample of patients 7.7-12.0 years of age, extraction of the primary canine had no significant effect on either eruption of the permanent canine or its position/inclination. However, extraction of the primary canine increased the eruption odds of the permanent canine significantly for patients older than nine or ten years of age, even though the data for this specific study sub-sample is very thin.

**Fig. 4:**
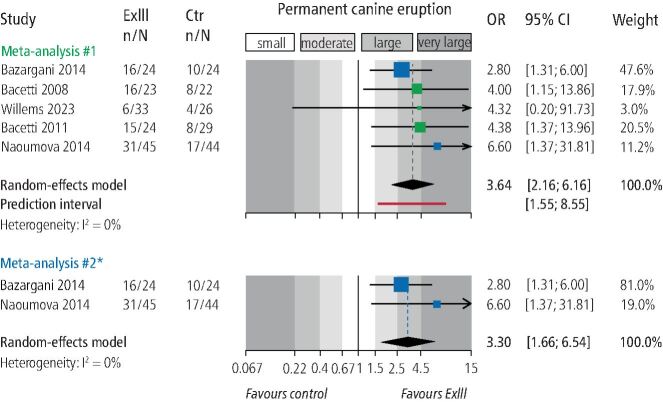
Forest plot on the effect of interceptive extraction of primary canine (versus no extraction) on the eruption of the impacted permanent canine of the same quadrant (CI = confidence interval; Ctr = control group [no extraction]; ExIII = extraction of the primary canine; n/N = events/sample; OR = odds ratio). *Meta-analysis #2 omits the three green-coloured studies that were included in meta-analysis #1

Meta-analysis of all five available studies (Table 3) indicated that extraction of the primary canine was associated with increased eruption odds for the permanent canine (five studies; OR = 3.6; 95% CI = 2.2-6.2; *p* <0.001; Fig. 4). As the prediction interval was situated completely to the right side of the forest plot, this benefit in the eruption odds of the permanent canine could be expected to be seen in all cases where the primary canine is extracted. However, it must be noted here that the two older trials from Italy^15^^,^^25^ present signs of baseline imbalances between compared groups, which raises questions about the success of the randomisation process (or indeed, if one was employed). Additionally, even though data from Willems *et al*.^26^ were modified to include only patients aged ten years or older, labially displaced canines might have also been included. Therefore, a sensitivity analysis was performed by excluding the two trials from Italy and the one from Belgium, which yielded mostly similar results (two trials; OR = 3.3; 95% CI = 1.7-6.5; *p* <0.001). Here, extraction of the primary canine was not consistently associated with an improvement in inclination of the permanent canine to the midline (two trials; MD = -2.83; *p* = 0.45). Evidence from single studies indicated that extraction of the primary canine was associated with reduced distance of the permanent canine cusp tip to the occlusal plane (MD: -1.1 mm) and improved odds that the permanent canine will ultimately end up in sectors one or two (OR = 3.6).

**Table 3:** Meta-analyses from included studies

**Outcome**	**Trials (3s)**	**Effect (95% CI)**	**p value**	**τ2 (95% CI)**	**I2 (95% CI)**	**Prediction**
**Extraction III versus control**						
3's eruption	5 (251)	OR 3.64 (2.16, 6.16)	<0.001	0 (0, 0.40)	0% (0%, 79%)	1.55, 8.55
3's eruption (modified)	2 (137)	OR 3.30 (1.66, 6.54)	<0.001	0 -	0% -	-
Alpha angle	2 (64)	MD -2.83 (-10.21, 4.55)	0.45	21.97 -	71% -	-
**Extraction III-IV versus extraction III**						
3's eruption	3 (221)	OR 2.50 (0.40, 15.56)	0.33	1.87 (0.03, 96.04)	74% (14%, 92%)	<0.01, >100.00
3's sector improvement by 1	3 (221)	OR 2.00 (1.13, 3.53)	0.01	0 (0, 0.08)	0% (0%, 90%)	0.05, 79.68
3's sector improvement by 2	3 (221)	OR 4.31 (0.70, 26.60)	0.12	1.34 (0, 76.85)	50% (0%, 85%)	<0.01, >100.00
3, permanent (displaced) canine; CI = confidence interval; III = deciduous canine; IV = deciduous first molar; MD = mean difference; OR = odds ratio.

## Evidence on extraction of the primary canine and primary first molar

Descriptives for the three included trials can be seen in Table 1. Two trials from the same research group originated from Italy^32^^,^^33^ and the third trial from Norway.^31^ All included both unilateral and bilateral cases aged 8.0-13.5, which means that patients only at risk of impaction were also included. The two trials from Italy included both palatally displaced and centrally (along the alveolar ridge) displaced canines, while the trial from Norway included only palatally displaced canines. Sample sizes ranged from 32-59 patients per trial (48-108 teeth per trial) and followed patients for 18 months or after canine eruption.

**Fig. 5:**
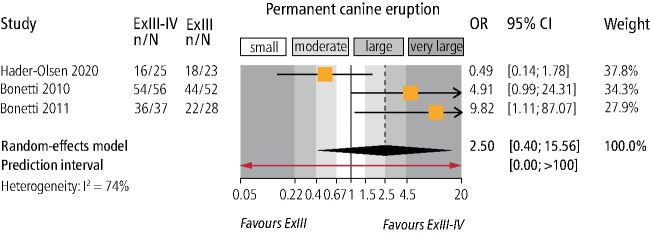
Forest plot on the effect of interceptive extraction of primary canine and the primary first molar (versus extraction of only the primary canine) on the eruption of the impacted permanent canine of the same quadrant (CI = confidence interval; ExIII = extraction of the primary canine; ExIII-IV = extraction of the primary canine and the primary first molar; n/N = events/sample; OR = odds ratio)

**Fig. 6:**
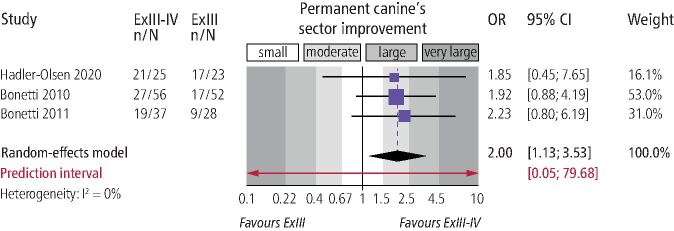
Forest plot on the effect of interceptive extraction of primary canine and the primary first molar (versus extraction of only the primary canine) on sector improvement of the impacted permanent canine of the same quadrant (CI = confidence interval; n/N = events/sample; OR = odds ratio)

Meta-analysis (Table 3) indicated that combined extraction of the primary canine and primary first molar was not associated with increased eruption odds for the permanent canine (three trials; OR = 2.5; 95% CI = 0.4-15.6; *p* = 0.33; Fig. 5). On the other hand, combined extraction of the primary canine-first molar was associated with increased odds that the permanent canine would improve its position by one sector on the panoramic radiograph (three trials; OR = 2.0; 95% CI = 1.1-3.5; *p* = 0.01; Fig. 6) compared to extraction of only the primary canine. However, no significant improvement by two sectors on the panoramic radiograph was seen (three trials; OR = 4.31; 95% CI = 0.7-26.6; *p* = 0.12). Finally, data from one trial^33^ indicated that combined extraction of the primary canine-first molar was associated with greater uprighting of the permanent canine (MDs of 7.2° and 8.7° for angle to the midline and lateral incisor, respectively) and of the first premolar (MD of 5.8° for angle to the midline) (see online Supplementary Information Table 4).

## How does current evidence inform clinical decision-making?

Current data would suggest that PDC eruption following interceptive extraction of the primary canine is unpredictable.

Due to limitations in study design of both retrospective and prospective investigations, there is a lack of robust data relating to how effective this intervention really is. Data from the few available randomised clinical trials indicates a potential benefit from interceptive extraction of the primary canine (alone or together with the primary first molar), but uncertainty exists around several issues. These include, among others, when the best time to carry out these extractions is, which patients might benefit most from these extractions, and what degree of displacement is likely to respond most favourably. It is also unclear from the current data whether extraction of the primary canine simply speeds up eruption of the PDC.

**Fig. 7:**
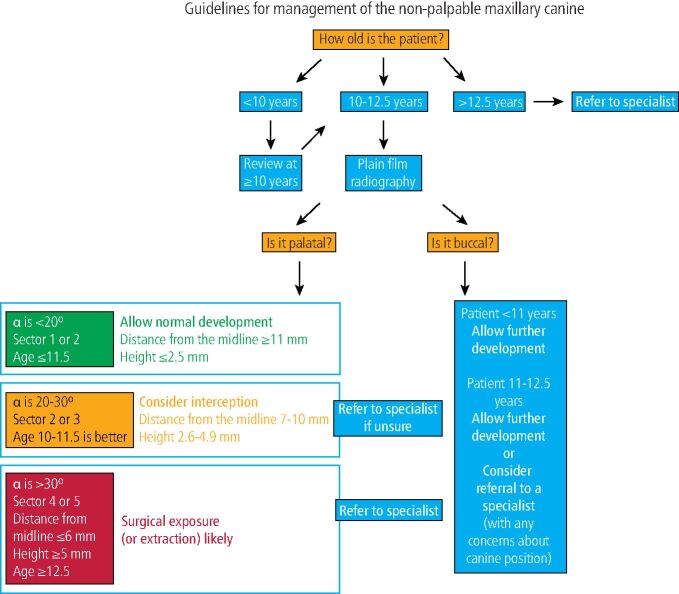
Guidelines for management of the non-palpable maxillary permanent canine. The vast majority of children would be expected to have maxillary canines in a buccal-palpable position at the age of 11 years, with these teeth erupted by the age of 12 years, although significant variation in eruption time can be seen.^45^ Guidelines relating to the PDC are based upon the current evidence base.^19^^,^^44^^,^^46^^,^^47^ If there is any concern about position or delayed eruption of a buccal-palpable maxillary canine, it is reasonable to undertake plain film radiographic investigation, particularly if the child is ≥12 years; however, guidance presented here for management of buccal positioned canines is the authors' opinion and not evidence-based. Collectively, these guidelines assume that there are no other issues with the developing malocclusion, particularly the presence of crowding in the maxillary arch. When there is any doubt, it is reasonable for the GDP to refer for specialist opinion at any age

So, how should we manage our patients (Fig. 7)? It could be suggested that if there is root resorption associated with the primary canine, and reasonable space in the maxillary arch, then there is minimal risk and very little to be potentially lost by extracting this tooth in attempting to self-correct a PDC - assuming the child will consent to extraction under local anaesthetic. It is clear from the literature that there does appear to be a ‘window' for this intervention. The age of patients included in studies ranges between 7.7-14 years. Extracting the primary canine beyond the age of 14 years is likely to be unsuccessful. Conversely, better outcomes in terms of normalising the PDC seem to be evident in younger patients (10-11 years old). Successful eruption of the PDC will also be hindered if there is a lack of space within the arch due to crowding. These points highlight the need for appropriate timing of this intervention and if there is any uncertainty, patients should be referred to a specialist orthodontist between the ages of 10-12 years for assessment and consideration of further treatment options.

A key predictor of a successful outcome appears to be the radiographic horizontal position of the canine crown in relation to the mid-sagittal plane (see Fig. 1). Based upon distance to the midline (whether this is classified as sectors or quantified in mm), clinicians may be able to assess if the maxillary canine will erupt normally or will require further intervention (Table 2).^17^^,^^19^^,^^43^

In summary, the closer the canine crown is to the midline (mid-sagittal plane), then the less likely it will erupt spontaneously after extraction of the primary canine (particularly those beyond sector three). Given the degree of uncertainty associated with this intervention, it would seem prudent to inform patients and their parents that eruption of the maxillary permanent canine should be monitored for 12 months following extraction of the primary canine. A follow-up period of 10-12 months has been suggested to evaluate the success of the interceptive extraction of primary canines, since the majority of permanent canines seem to erupt without assistance within 12 months of the intervention (even though some might take up to 22 months or more to erupt without further intervention).^44^ It is also important that space at the extraction site is maintained, especially for longer follow-up periods, otherwise it might continuously decrease.^17^ If after the observation period, the canine has not erupted, then surgical exposure and orthodontic alignment of the canine could be required as part of the overall management of the malocclusion.

## Conclusions

This review has highlighted the current evidence base relating to the effectiveness of primary maxillary canine extraction to normalise the position of a PDC. The success of this intervention is unpredictable, but a favourable outcome can be achieved if treatment is commenced between the ages of 10-13 years, the patient is in the mixed dentition, there is space for the permanent canine within the dental arch, and radiographically, the permanent canine crown is not close to the mid-sagittal plane/midline.

## Supplementary Information

Supplementary Information (PDF 200KB)
