# Supplementary material for: The palatal displaced maxillary canine: early diagnosis and interceptive correction - a guideline for the general dental practitioner
Source: Br Dent J. 2025 Oct 10;239(7):463–70. doi: 10.1038/s41415-025-8892-z (PMC12513824; doi:10.1038/s41415-025-8892-z)
Supplement: Supplementary file 1 — Supplementary Information (PDF 200KB) [file 41415_2025_8892_MOESM1_ESM.pdf]

SI Table 1. Communication attempts for additional data (raw dataset or adjusted-for-clustering estimates) with their status

Trial	Corresponding author	Status
Extraction III versus control		
Baccetti 2008 [14]	Tiziano Baccetti	No request made
Baccetti 2011 [24]	Tiziano Baccetti	No request made
Bazargani 2014 [16]	Farhan Bazargani	Request made; sent estimated odds with 95% confidence interval for outcome of eruption after adjusting for clustering within patients
Naoumova 2014$_{coll}$ [26]	Julia Naoumova	Request made; sent dataset of patients with demographics and outcome (eruption)
Willems 2023 [25]	Guy Willems	Request made; sent full dataset of all patients with demographics and outcomes (eruption; canine inclination; canine position)
Extraction III-IV versus extraction III		
Alessandri Bonetti 2010 [32]	Giulio Alessandri Bonetti	Request made; no response
Alessandri Bonetti 2011 [31]	Giulio Alessandri Bonetti	Request made; no response
Hadler-Olsen 2020$_{coll}$ [30]	Sigurd Hadler-Olsen	Request made; no response

14. Baccetti T, Leonardi M, Armi P. A randomized clinical study of two interceptive approaches to palatally displaced canines. *Eur J Orthod*. 2008; **30:** 381-5.
24. Baccetti T, Sigler LM, McNamara JA, Jr. An RCT on treatment of palatally displaced canines with RME and/or a transpalatal arch. *Eur J Orthod* 2011; **33:** 601-7.
16. Bazargani F, Magnuson A, Lennartsson B. Effect of interceptive extraction of deciduous canine on palatally displaced maxillary canine: a prospective randomized controlled study. *Angle Orthod* 2014; **84:** 3-10.
26. Naoumova J. Interceptive Treatment Of Palatally Displaced Canines Studies of treatment effect and patients' perception and methodological evaluation of 3D measurements of CBCT. Doctoral thesis, 2014, University of Gothenburg Sahlgrenska Academy http://hdlhandlenet/2077/36747. 2014.
25. Willems G, Butaye C, Raes M, Zong C, Begnoni G, Cadenas de Llano-Perula M. Early prevention of maxillary canine impaction: a randomized clinical trial. *Eur J Orthod* 2023; **45:** 359-69.
32. Alessandri Bonetti G, Incerti Parenti S, Zanarini M, Marini I. Double vs single primary teeth extraction approach as prevention of permanent maxillary canines ectopic eruption. *Pediatr Dent* 2010; **32:** 407-12.
31. Alessandri Bonetti G, Zanarini M, Incerti Parenti S, Marini I, Gatto MR. Preventive treatment of ectopically erupting maxillary permanent canines by extraction of deciduous canines and first molars: A randomized clinical trial. *Am J Orthod Dentofacial Orthop* 2011; **139:** 316-23.
30. Hadler-Olsen S, Sjogren A, Steinnes J, Dubland M, Bolstad NL, Pirttiniemi P, et al. Double vs single primary tooth extraction in interceptive treatment of palatally displaced canines. *Angle Orthod* 2020; **90:** 751-7.

SI Table 2a. Re-analysis of the Naoumova et al [Naoumova et al., 2014] [26] trial; factors affecting the eruption of palatally displaced canines

		**Unilateral**			**Bilateral**			**Uni-/bi-lateral**	
**Factor**	**Category**	**OR (95% CI)**	**P**		**OR (95% CI)**	**P**		**OR (95% CI)**	**P**
Age	Per year	0.69 (0.38, 1.25)	0.22		0.80 (0.44, 1.45)	0.47		0.74 (0.48, 1.14)	0.17
Sex	Female	Reference			Reference			Reference	
	Male	2.36 (0.65, 8.51)	0.19		1.2 (0.46, 3.14)	0.71		1.59 (0.74, 3.42)	0.23
alpha angle	Per degree	0.28 (0.11, 0.71)	0.007		0.47 (0.25, 0.87)	0.01		0.40 (0.22, 0.71)	0.002
alpha category	<30 degrees	Reference			Reference			Reference	
	≥ 30 degrees	<0.01 (<0.01, <0.01)	<0.001		<0.01 (<0.01, <0.01)	<0.001		<0.01 (<0.01, <0.01)	<0.001
d distance	Per mm	1.17 (0.92, 1.49)	0.20		0.98 (0.74, 1.29)	0.88		1.06 (0.89, 1.29)	0.50
m distance	Per mm	0.93 (0.73, 1.19)	0.56		1.06 (0.85, 1.31)	0.62		1.00 (0.85, 1.18)	0.99
Sectors	1-2	Reference			Reference			Reference	
	≥ 3	0.02 (<0.01, 0.13)	<0.001		0.04 (0.01, 0.19)	<0.001		0.03 (0.01, 0.10)	<0.001
Root development	Per stage	0.47 (0.20, 1.10)	0.08		0.59 (0.30, 1.14)	0.11		0.53 (0.31, 0.90)	0.02

CI, confidence interval; OR, odds ratio.

26. Naoumova J. Interceptive Treatment Of Palatally Displaced Canines Studies of treatment effect and patients' perception and methodological evaluation of 3D measurements of CBCT. Doctoral thesis, 2014, University of Gothenburg Sahlgrenska Academy http://hdlhandlenet/2077/36747. 2014.

SI Table 2b. Re-analysis of the Naoumova et al [Naoumova et al., 2014] [26] trial; identification of confounders to the effect of primary canine extraction on the eruption of palatally displaced canines with the Change-In-Estimate method

	**Unilateral**				**Bilateral**				**Uni-/Bi-later**		
**Covariate**	**Coefficient**	**% CIE**	**Selected**		**Coefficient**	**% CIE**	**Selected**		**Coefficient**	**% CIE**	**Selected**
- (crude)	0.8109	-	-		1.7430	-	-		1.2576	-	-
Age	1.0020	23.6%	Yes		1.7708	1.6%	No		1.3637	8.4%	No
Sex	0.7802	-3.8%	No		1.7479	0.3%	No		1.2570	0.0%	No
alpha angle	18.0225	2122.5%	Yes		2.9918	71.7%	Yes		3.9830	216.7%	Yes
alpha category	0.8755	8.0%	No		1.6740	-4.0%	No		1.2294	-2.2%	No
d distance	1.0398	28.2%	Yes		1.7900	2.7%	No		1.2831	2.0%	No
m distance	0.7733	-4.6%	No		1.7487	0.3%	No		1.2667	0.7%	No
Sector	1.8801	131.8%	Yes		1.9526	12.0%	No		1.8252	45.1%	Yes
Root development	0.9258	14.2%	No		1.8441	5.8%	No		1.3649	8.5%	No

CIE, change in estimate .

26. Naoumova J. Interceptive Treatment Of Palatally Displaced Canines Studies of treatment effect and patients' perception and methodological evaluation of 3D measurements of CBCT. Doctoral thesis, 2014, University of Gothenburg Sahlgrenska Academy http://hdlhandlenet/2077/36747. 2014.

SI Table 2c. Re-analysis of the Naoumova et al [Naoumova et al., 2014] [26] trial; effect of primary canine extraction on the eruption of palatally displaced canines with crude and adjusted-for-confounders analysis

	**Unilateral**			**Bilateral**			**Uni-/Bi-lateral**	
**Model adjustment**	**OR (95% CI)**	**P**		**OR (95% CI)**	**P**		**OR (95% CI)**	**P**
None (crude)	2.25 (0.68, 7.47)	0.19		5.71 (1.30, 25.07)	0.02		3.52 (1.39, 8.92)	0.008
Age	2.72 (0.77, 9.59)	0.12		NT			NT	
alpha angle	>100 (<0.01, >100)	0.73		19.92 (0.10, >100)	0.27		53.68 (0.12, >100)	0.20
alpha category	2.4 (0.60, 9.63)	0.22		5.33 (1.00, 28.55)	<0.001		3.42 (1.19, 9.85)	0.02
d distance	2.83 (0.78, 10.28)	0.11		NT				
Sector	6.55 (0.70, 61.73)	0.10		NT			6.20 (1.45, 26.62)	0.01

CI, confidence interval; NT, not tested; OR, odds ratio.

26. Naoumova J. Interceptive Treatment Of Palatally Displaced Canines Studies of treatment effect and patients' perception and methodological evaluation of 3D measurements of CBCT. Doctoral thesis, 2014, University of Gothenburg Sahlgrenska Academy http://hdlhandlenet/2077/36747. 2014.

SI Table 3a. Re-analysis of the Willems et al [Willems et al., 2023] [25] trial; identification of confounders to the effect of primary canine extraction on the eruption of labially / palatally displaced canines with the Change-In-Estimate method

	**Eruption**			**3-ML (º)**			**3-4 (º)**			**3-ML (mm)**			**3-4 (mm)**		
**Covariate**	**b**	**% CIE**	**Sel**	**b**	**% CIE**	**Sel**	**b**	**% CIE**	**Sel**	**b**	**% CIE**	**Sel**	**b**	**% CIE**	**Sel**
- (crude)	0.2007	-	-	0.7186	-	-	4.6370	-	-	-0.2967	-	-	-0.6720	-	-
Age	0.1151	-42.6%	Yes	0.7131	-0.76%	No	5.1715	11.53%	No	-0.3057	3.04%	No	-0.6841	1.80%	No
Sex	0.1923	-4.2%	No	0.7242	0.78%	No	4.1592	-10.30%	No	-0.2588	-12.77%	No	-0.7148	6.36%	No
Follow-up duration	0.8793	338.2%	Yes	0.8127	13.10%	No	4.7549	2.54%	No	-0.3299	11.18%	No	-0.9846	46.52%	Yes
Sector	0.2780	38.6%	Yes	0.4790	-33.34%	Yes	2.5928	-44.09%	Yes	-0.2032	-31.50%	Yes	-1.1716	74.33%	Yes
Initial inclination to ML	0.4593	128.9%	Yes	NT	-	-	0.5270	-88.64%	Yes	-0.0856	-71.16%	Yes	-0.7301	8.65%	No
Initial inclination to PM1	0.1860	-7.3%	No	-0.0092	-101.28%	Yes	NT	-	-	-0.7472	151.85%	Yes	-0.1828	-72.80%	Yes
Initial distance to ML	0.2376	18.4%	No	0.6987	-2.77%	No	4.5831	-1.16%	No	NT	-	-	-0.3847	-42.76%	Yes
Initial distance to occlusal plane	-0.2157	-207.5%	Yes	0.4721	-34.30%	Yes	4.5074	-2.79%	No	-0.4481	51.03%	Yes	NT	-	-

CIE, change in estimate; Sel, selected.

25. Willems G, Butaye C, Raes M, Zong C, Begnoni G, Cadenas de Llano-Perula M. Early prevention of maxillary canine impaction: a randomized clinical trial. *Eur J Orthod* 2023; **45:** 359-69.

SI Table 3b. Re-analysis of the Willems et al [Willems et al., 2023] [25] trial; effect of primary canine extraction on the eruption of labially / palatally displaced canines with crude / adjusted-for-confounders analysis

		**All patients (n=31)**			**>9 years (n=11)**			**>10 years (n=8)**	
**Outcome**	**Model adjustment**	**OR (95% CI)**	**P**		**OR (95% CI)**	**P**		**OR (95% CI)**	**P**
Eruption	None (crude)	1.22 (0.18, 8.17)	0.84		>100 (>100, >100)	<0.001		>100 (>100, >100)	<0.001
	Age	1.12 (0.15, 8.20)	0.91		NT			NT	
	Follow-up duration	2.41 (0.70, 8.29)	0.16		NT			NT	
	Sector	1.32 (0.20, 8.73)	0.77		NT			NT	
	Initial inclination to ML	1.58 (0.22, 11.50)	0.65		NT			NT	
	Initial distance to occlusal plane	0.81 (0.13, 4.93)	0.82		NT			NT	
	**Model adjustment**	**b (95% CI)**	**P**		**b (95% CI)**	**P**		**b (95% CI)**	**P**
3-ML (º)	None (crude)	0.72 (-0.43, 1.86)	0.22		-0.13 (-0.52, 0.26)	0.50		-0.03 (-0.52, 0.45)	0.89
	Sector	0.48 (-0.42, 1.38)	0.30						
	Inc341	-0.01 (-0.67, 0.65)	0.98						
	Disop1	0.47 (-0.33, 1.28)	0.25						
3-4 (º)	None (crude)	4.64 (-3.32, 12.60)	0.25		-4.42 (-12.68, 3.84)	0.29		-2.09 (-9.32, 5.14)	0.57
	Sector	2.59 (-3.88, 9.06)	0.43						
	Initial inclination to ML	0.53 (-5.33, 6.39)	0.86						
3-ML (mm)	None (crude)	-0.30 (-1.56, 0.96)	0.64		-1.06 (-3.64, 1.52)	0.42		-1.49 (-5.66, 2.68)	0.48
3-4 (mm)	None (crude)	-0.67 (-3.18, 184)	0.60		1.30 (-1.96, 4.56)	0.43		0.98 (-3.25, 5.21)	0.65
	Follow-up	-0.98 (-3.37, 1.40)	0.42						
	Sector	-1.17 (-3.63, 1.29)	0.35						
	Initial inclination to ML	-0.18 (-2.47, 2.10)	0.88						
	Initial distance to ML	-0.38 (-2.70, 1.93)	0.75						

CI, confidence interval; OR, odds ratio.

25. Willems G, Butaye C, Raes M, Zong C, Begnoni G, Cadenas de Llano-Perula M. Early prevention of maxillary canine impaction: a randomized clinical trial. *Eur J Orthod* 2023; **45:** 359-69.

SI Table 4. Results of outcomes assessed by individual studies that could not be meta-analysed

Nr	Comparison	Trial	Outcome	Effect	95% CI	P	Significant at 5%	Clinically relevant*
1	Ex III versus control	Naoumova 2014 [26]	Canine cusp tip-dental arch plane; 12 months (mm)	MD -1.10	-1.80, -0.40	0.002	Yes	**Yes**
2	Ex III versus control	Bazargani 2014 [16]	Canine cusp tip-dental arch plane; 18 months (mm)	MD -2.20	-4.40, 0.60	0.06	No	-
3	Ex III versus control	Naoumova 2014 [26]	Canine cusp tip-dental arch plane; 6 months (mm)	MD -0.30	-1.20, 0.60	0.51	No	-
4	Ex III versus control	Naoumova 2014 [26]	Canine cusp tip-midline; 12 months (mm)	MD -0.62	-1.14, -0.10	0.01	Yes	No
5	Ex III versus control	Naoumova 2014 [26]	Canine cusp tip-midline; 6 months (mm)	MD 1.70	0.90, 2.50	<0.001	Yes	No
6	Ex III versus control	Bazargani 2014 [16]	Canine in zone 1	OR 2.80	0.87, 9.06	0.09	No	-
7	Ex III versus control	Bazargani 2014 [16]	Canine in zones 1-2	OR 3.55	1.04, 12.07	0.04	Yes	**Yes**
8	Ex III versus control	Bazargani 2014 [16]	Canine in zones 1-2-3	OR 4.53	0.83, 24.64	0.08	No	-
9	Ex III versus control	Naoumova 2014 [26]	Canine root apex-dental arch plane; 12 months (mm)	MD 0.80	0.20, 1.40	0.009	Yes	No
10	Ex III versus control	Naoumova 2014 [26]	Canine root apex-dental arch plane; 6 months (mm)	MD 0.20	-0.40, 0.80	0.51	No	-
11	Ex III versus control	Willems 2023 [25]	Canine-1st premolar angle (º)	MD -2.09	-9.32, 5.14	0.57	No	-
12	Ex III versus control	Willems 2023 [25]	Canine-1st premolar distance (mm)	MD 0.98	-3.25, 5.21	0.65	No	-
13	Ex III versus control	Willems 2023 [25]	Canine-ML distance (mm)	MD -1.49	-5.66, 2.68	0.48	No	-
14	Ex III versus control	Naoumova 2014 [26]	Eruption time (months)	MD 0.11	-3.53, 3.75	0.95	No	-
15	Ex III versus control	Naoumova 2014 [26]	Mesioangular angle; 12 months (º)	MD -2.80	-5.40, -0.20	0.03	Yes	No
16	Ex III versus control	Naoumova 2014 [26]	Mesioangular angle; 6 months (º)	MD -2.60	-5.10, -0.10	0.04	Yes	No
17	Ex III versus control	Naoumova 2014 [26]	Root resorption	OR 0.52	0.19, 1.41	0.20	No	-
18	Ex III versus control	Naoumova 2014 [26]	Sagittal angle; 12 months (º)	MD 1.70	-1.30, 4.70	0.27	No	-
19	Ex III versus control	Naoumova 2014 [26]	Sagittal angle; 6 months (º)	MD 0.70	-2.20, 3.60	0.64	No	-
20	Ex III versus control	Naoumova 2014 [26]	Vertical position; 12 months (mm)	MD 1.20	0.40, 2.00	0.003	Yes	**Yes**
21	Ex III versus control	Naoumova 2014 [26]	Vertical position; 6 months (mm)	MD 0.90	0.20, 1.60	0.01	Yes	No
22	Ex III-IV versus Ex III	Hadler-Olsen 2020 [30]	Canine sector deterioration by 1	OR 0.91	0.12, 7.08	0.93	No	-
23	Ex III-IV versus Ex III	Hadler-Olsen 2020 [30]	Canine sector improvement by 3	OR 0.44	0.04, 5.18	0.51	No	-
24	Ex III-IV versus Ex III	Alessandri Bonetti 2010 [32]	Change in alpha angle (º)	MD 7.20	4.06, 10.34	<0.001	Yes	**Yes**
25	Ex III-IV versus Ex III	Alessandri Bonetti 2010 [32]	Change in beta angle (º)	MD 8.70	4.26, 13.14	<0.001	Yes	**Yes**
26	Ex III-IV versus Ex III	Alessandri Bonetti 2010 [32]	Change in pi angle (º)	MD 5.80	3.05, 8.55	<0.001	Yes	**Yes**

CI, confidence interval; Ex, extraction; III, deciduous canine; IV, deciduous 1st molar; MD, mean difference; OR, odds ratio.
* clinically relevant effect (judged as >1/2 standard deviation of the control at baseline);

26. Naoumova J. Interceptive Treatment Of Palatally Displaced Canines Studies of treatment effect and patients' perception and methodological evaluation of 3D measurements of CBCT. Doctoral thesis, 2014, University of Gothenburg Sahlgrenska Academy http://hdlhandlenet/2077/36747. 2014.
16. Bazargani F, Magnuson A, Lennartsson B. Effect of interceptive extraction of deciduous canine on palatally displaced maxillary canine: a prospective randomized controlled study. *Angle Orthod* 2014; **84:** 3-10.
25. Willems G, Butaye C, Raes M, Zong C, Begnoni G, Cadenas de Llano-Perula M. Early prevention of maxillary canine impaction: a randomized clinical trial. *Eur J Orthod* 2023; **45:** 359-69.
30. Hadler-Olsen S, Sjogren A, Steinnes J, Dubland M, Bolstad NL, Pirttiniemi P, et al. Double vs single primary tooth extraction in interceptive treatment of palatally displaced canines. *Angle Orthod* 2020; **90:** 751-7.
32. Alessandri Bonetti G, Incerti Parenti S, Zanarini M, Marini I. Double vs single primary teeth extraction approach as prevention of permanent maxillary canines ectopic eruption. *Pediatr Dent* 2010; **32:** 407-12.
